# Effects of high frequency loading on RANKL and OPG mRNA expression in ST-2 murine stromal cells

**DOI:** 10.1186/1471-2474-10-109

**Published:** 2009-09-04

**Authors:** Chi Hyun Kim, Kyung Hwan Kim, Christopher R Jacobs

**Affiliations:** 1 Department of Biomedical Engineering, Yonsei University, Wonju, Gangwon Do, Korea; 2 Department of Biomedical Engineering, Columbia University, New York, NY, USA; 3 Department of Mechanical Engineering, Stanford University, Stanford, CA, USA

## Abstract

**Background:**

Oscillatory fluid flow (OFF)-induced shear stress leads to positive bone remodeling through pro-formative and anti-resorptive effects on bone cells. In this study, the effects of high frequency OFF on expression of receptor activator of NF-κB ligand (RANKL) and osteoprotegerin (OPG), two important regulators of osteoclast differentiation, were investigated.

**Methods:**

Cells were exposed to 1 Pa peak shear stress using three loading frequencies (1, 10, and 20 Hz) widely employed in cell, animal, and clinical studies of bone remodeling. Two separate experiments were performed where either the total number of cycles (3600 cycles) or the total loading time (60 min) was kept constant. Real-time RT-PCR was used to quantify mRNA levels of RANKL, OPG.

**Results:**

3600 cycles of OFF at 1 Hz and 10 Hz loading decreased RANKL/OPG ratio. Interestingly, these results were due to different mechanisms where at 1 Hz the decrease was due to an increase in OPG mRNA, whereas at 10 Hz the decrease was due to a decrease in RANKL mRNA.

**Conclusion:**

Although high frequency OFF does not appear to further enhance the decrease in the RANKL/OPG ratio, these results suggest a potential to differentially control the change in either RANKL or OPG mRNA expression by applying different loading frequencies.

## Background

It is widely accepted that mechanical loading is a critical factor that regulates bone metabolism. Interestingly, the temporal pattern of loading is known to be important in the response of bone. Specifically, the anabolic responses that occur when loading is applied in a dynamic fashion do not occur with static loading.[1] Within the dynamic loading regime, mechanical loading parameters (i.e., strain magnitude, loading frequency, strain waveform, and number of loading cycles) can have an important effect of outcome. [2-4]

Significance of loading frequency has been studied where an optimal loading frequency within the range of 5 Hz to 30 Hz has been suggested using both animal and clinical studies. [5-7] In vitro studies using loading frequencies of 1 Hz to 9 Hz have found that increasing pulsatile fluid flow frequency results in increased production of nitric oxide but not prostaglandin E2, two signaling molecules that play important roles in bone remodeling.[8]

A potent cellular physical signal in the regulation of bone metabolism is loading-induced fluid flow. [8-13] When bone is functionally loaded, fluid is forced out of regions of high compressive strains and then returns when the load is removed resulting in bone cells being exposed to a dynamic oscillating fluid flow.[14] In vitro studies show that cells are responsive to physiological levels of OFF. [15-17] suggesting that OFF may be an appropriate physical signal to study mechanotransduction in bone cells.[12,13,18]

Nuclear factor kappa B (NF-κB) ligand (RANKL) and osteoprotegerin (OPG) are two molecules expressed by pre-osteoblastic cells that regulate osteoclast formation.[19,20] RANKL is a membrane bound protein that stimulates the osteoclast precursors to commit to the osteoclastic phenotype by binding to its receptor (RANK) on the surface of osteoclast precursors.[21] RANKL is expressed when stimulated by various hormones and cytokines including vitamin D.[22,23] OPG is a decoy receptor that competes with RANK for binding of RANKL.[24,25]

In a previous study, ST-2 murine stromal cells exposed to OFF resulted in a dose-dependent increase in OPG and decrease in RANKL mRNA levels.[13] Furthermore, co-culture of RAW264.7 monocytes with ST-2 cells exposed to OFF led to a significant decrease in the total number of osteoclasts generated as well as in osteoclastic resorptive activity compared to co-culture with ST-2 cells not exposed to OFF. These results suggest that the amount of bone resorbed is dictated by the balance between RANKL and OPG.[26,27] However, the effects of high loading frequencies on stromal/osteoblastic cells that regulate osteoclastic differentiation have not been examined. It is also critical to understand the individual response of RANKL and OPG at various loading frequencies as well as to examine the existence of an optimal loading frequency that may lead to maximal suppression of bone resorption.

In this study, we investigated the effects of high frequency loading on the gene expression of RANKL and OPG using a physiological cell-level mechanical signal. Specifically, oscillatory fluid flow-induced shear stress was applied to ST-2 cells using 1 Hz, 10 Hz and 20 Hz loading frequencies (loading frequencies widely employed in cell, animal, and clinical studies for anabolic responses). Individual RANKL and OPG mRNA expression levels as well as the ratio between RANKL and OPG mRNA level at each loading frequency were quantified.

## Methods

### Cell Culture and Oscillatory Fluid Flow

ST-2 (Riken, Japan) murine bone marrow stromal cells were cultured on tissue culture dishes in alpha-MEM (Invitrogen, Carlsbad, CA) with 10% FBS (HyClone, UT) and 1% penicillin/streptomycin (Invitrogen, CA). Cells were placed in a humidified incubator at 37 C and 5% CO_2_. Once the cells reached 80% confluence, they were subcultured on glass slides (76 × 48 × 1 mm) at a density of approximately 2 × 10^5 ^cells/cm^2^. 10 nM 1α,25-dihydroxyvitamin D_3 _(Fluka, Switzerland) was added to induce expression of RANKL. Cells were grown for an additional 48 hours at which time they had reached approximately 90% confluence.

The slides were then placed in custom-built sterile parallel plate flow chambers under sterile conditions. Oscillatory fluid flow was driven by a Hamilton glass syringe connected in series with rigid walled tubing and a parallel plate flow chamber as previously described.[18] The syringe was mounted in and driven by a feedback-controlled linear electromagnetic actuator that can deliver a precise flow rate (EnduraTec, MN). The flow rate was monitored using an ultrasonic flow meter (Transonic Systems Inc., NY). The flow rate was selected to yield a peak shear stress of ± 1 Pa using a sinusoidal waveform. Flow media for all OFF experiments consisted of alpha-MEM with 10% FBS and 1% penicillin/streptomycin. The parallel plate flow chambers were placed in the humidified incubator for the entire duration of loading. Control cells were also subcultured on glass slides, exposed to 1α,25-dihydroxyvitamin D_3_, and placed in the parallel plate flow chamber for the same time period as the treated cells but were not exposed to fluid flow.

### Constant Loading Cycle

Cells were exposed to a total of 3600 cycles of OFF using frequencies of 1, 10, or 20 Hz. This resulted in loading durations of 60 min (1 Hz), 6 min (10 Hz), and 3 min (20 Hz). Each group consisted of 4 samples.

### Constant Loading Time

Cells were exposed to a total of 60 min OFF using various frequencies. Therefore, cells were exposed to either 3600 cycles (1 Hz), 36000 cycles (10 Hz), or 72000 cycles (20 Hz) of OFF. Each group consisted of 4 samples.

### RNA Isolation and Real-Time RT-PCR

Immediately after the end of the OFF experiment, cells were lysed and total RNA isolated using Tri-Reagent (Sigma, MO). The 260/280 absorbance ratio was measured for verification of the purity of RNA. Analysis by quantitative real-time RT-PCR (Perkin Elmer Prism 7900, Applied Biosystems, Foster City, CA) was conducted to determine the relative steady state mRNA levels of RANKL and OPG (Mm00441908_m1 and Mm00435452_m1, Taqman Gene Expression Assays, Applied Biosystems, Foster City, CA). Additionally, rRNA for the housekeeping gene 18S (4310893E, Taqman Gene Expression Assays, Applied Biosystems, Foster City, CA) was analyzed for each sample. Each RNA sample was analyzed in triplicate.

### Statistics

RANKL and OPG gene expression levels were normalized against 18S rRNA assayed in the same sample tube. Statistical changes in gene expression were analyzed using Student's t-test with a significant difference assumed at p < 0.05. Error bars represent standard error of the mean.

## Results

### Constant Loading Cycle

**Figure 1:**
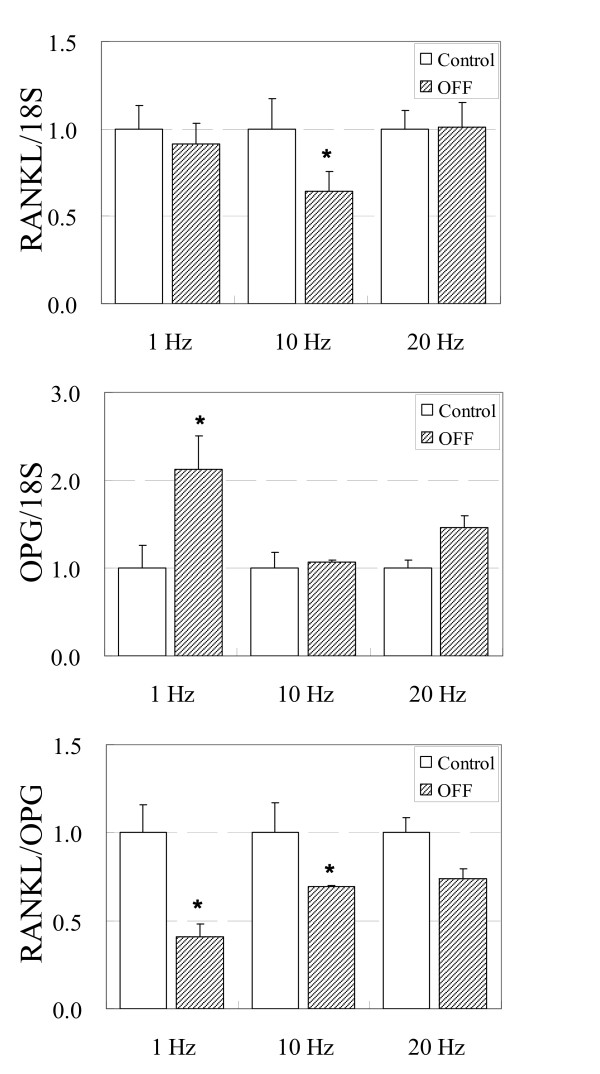
**RANKL, OPG, and RANKL/OPG mRNA levels in ST-2 murine stromal cells after 3600 cycles of OFF**. Loading frequencies of 1, 10, or 20 Hz were used. This resulted in loading durations of 60 min (1 Hz), 6 min (10 Hz), and 3 min (20 Hz). N = 4 for each group.

Immediately after application of 3600 cycles of OFF, the RANKL/OPG ratio significantly decreased in 1 Hz (60 min) and 10 Hz (6 min) loading groups compared to no flow controls (p < 0.05) (Figure 1). The RANKL/OPG ratio decreased by approximately 60% using a loading frequency of 1 Hz and approximately 30% using a loading frequency of 10 Hz. Interestingly though, the decreases in RANKL/OPG ratio at these two loading frequencies (i.e., 1 Hz and 10 Hz) compared to no flow controls resulted occurred in different ways. For 1 Hz loading, RANKL mRNA expression displayed no change compared to no flow controls whereas OPG mRNA expression significantly increased by over 2-fold leading to an overall significant decrease in RANKL/OPG ratio. On the other hand, for 10 Hz loading, RANKL mRNA expression significantly decreased by 35% whereas OPG mRNA expression showed no change compared to no flow controls. This decrease in RANKL resulted in an overall significant decrease in RANKL/OPG ratio.

In contrast to our results at 1 Hz and 10 Hz loading frequencies, application of 3600 cycles of OFF using a loading frequency of 20 Hz (3 min loading) did not result in a significant difference compared to no flow control groups (p > 0.05) (Figure 1). At this loading frequency, both RANKL and OPG mRNA levels remained unchanged compared to no flow controls.

### Constant Loading Time

**Figure 2:**
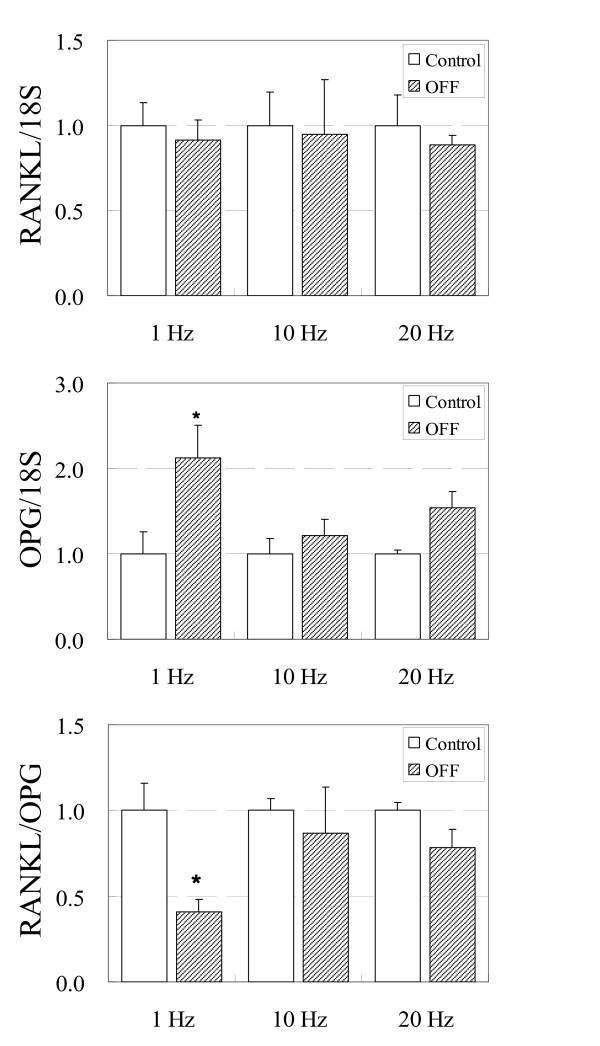
**RANKL, OPG, and RANKL/OPG mRNA levels in ST-2 murine stromal cells after 60 min OFF**. Loading frequencies of 1, 10, or 20 Hz were used. This resulted in loading cycles of 3600 cycles (1 Hz), 36000 cycles (10 Hz), or 72000 cycles (20 Hz) of OFF. N = 4 for each group.

Application of OFF for a total of 60 min resulted in a significant decrease in RANKL/OPG ratio compared to corresponding no flow controls at 1 Hz (3600 cycles) loading frequency only (p < 0.05) (Figure. 2). At higher loading frequencies of 10 Hz (36000 cycles) and 20 Hz (72000 cycles), which resulted in significantly more loading cycles, RANKL/OPG ratio showed a trend of decrease compared to no flow controls but neither group resulted in significant differences (p > 0.05). Sixty-minute loading at these higher frequencies did not result in significant differences in RANKL and OPG mRNA expression compared to no flow control groups.

## Discussion

In this study, we investigated the effects of various loading frequencies previously reported to produce an anabolic effect on bone remodeling in animal and clinical studies. An established in vitro cell loading system was used to deliver 1 Hz, 10 Hz, and 20 Hz loading frequencies.[12,13,18,28] Specifically, oscillatory fluid flow (OFF), a cell-level physical signal that occurs with cyclic tissue loading, was applied while assaying changes in the expression of RANKL and OPG mRNA, two important regulators in bone resorption, in murine stromal cells.

Similar to a previous dose-response study, 3600 cycles (i.e., 1 Hr) of OFF using a loading frequency of 1 Hz resulted in a significant decrease in RANKL/OPG ratio.[13] Once again, this decrease was due to a significant 2-fold increase in OPG. On the other hand, 3600 cycles of 1 Hz OFF did not have an effect on RANKL mRNA expression, similar to the previous study. When 3600 cycles of OFF was applied at 10 Hz frequency, the RANKL/OPG ratio was also significantly decreased (approximately 30% decrease) although the level of decrease was not as dramatic as the 1 Hz loading (approximately 60% decrease). In contrast to the results of 1 Hz loading, this decrease in RANKL/OPG ratio was due to not an increase in OPG mRNA level but a significant decrease in RANKL mRNA level. When 3600 cycles of OFF was applied at 20 Hz frequency, RANKL and OPG mRNA expressions did not change and subsequently the RANKL/OPG ratio remained unchanged. These results are consistent with in vivo studies that show the most efficient bone formation occurring at 5-10 Hz frequencies.[6] The fact that 10 Hz loading decreases RANKL/OPG ratio via a different mechanism compared to 1 Hz frequency loading suggests that there may be an optimal loading frequency in the range of 1 Hz and 10 Hz where the synergistic effect between the decrease in RANKL and increase in OPG is maximal.[6]

Other mechanical stimuli have also been examined in terms of their anti-resorptive effects. [29-33] For example, Rubin et al. have shown that substrate deformation using 0.16 Hz loading leads to a drop in RANKL/OPG ratio due to a change in RANKL expression but not OPG.[30] On the other hand, Kusumi et al. showed an increase in OPG synthesis and a decrease in RANKL mRNA expression in osteoblasts exposed to 0.2 Hz to 0.3 Hz cyclic tensile strain.[29] However, the frequency ranges adopted in these previous studies are well below the frequency ranges of the activities of daily living and those generally used in animal and clinical bone remodeling studies.[6,34,35] Thus, it is not clear whether the differences observed in this study are the result of a different simulation frequency or a different mechanical stimulus. It would be interesting to examine the individual RANKL and OPG outcomes using a higher frequency substrate deformation to determine if the differential effects observed in this study (i.e., cells exposed to fluid-induced shear stress) is also observed in cells exposed to mechanical strain.

Results from this study suggest the possible use of multiple loading frequencies in mechanically stimulating bone formation. For example, short bouts of high frequency loading (e.g., 10 Hz) may be incorporated into low frequency (e.g., 1 Hz) loading regimens for treatment of osteoporosis. Furthermore, different loading frequencies may be applied depending on the patients' conditions in order to differently control expression of RANKL and OPG gene expression.

Although 6 min (3600 cycles) of 10 Hz loading decreased the RANKL/OPG ratio by approximately 30%, when loading was continued for 1 Hr, the drop in ratio was recovered and resulted in a non-significant change compared to no flow controls. Similarly, applying 20 Hz frequency loading for 1 Hr did not lead to changes in RANKL, OPG and RANKL/OPG ratio. These results indicate that RANKL downregulation and OPG upregulation due to loading-induced shear stress are recovered to baseline after longer loading durations. Hence, unnecessarily prolonged loading appears to be ineffective and it is important to determine a loading duration that produces the greatest anti-resorptive effects. This optimal loading duration is also suggested in cellular and animal studies which show that a single continuous long-duration loading is less effective compared to multiple short bout loading of equal total loading duration.[12,36] It is possible that although high frequency stimulations result in more frequent loading events, it appears that by not allowing the cells to recover, the potential anti-resorptive effects of these may be lost. It is also possible that strain history is integrated into the cellular memory so that the reference state for bone cell response is constantly changing as described in vivo.[37] As a result, prolonged loading without adjusting the loading pattern might result in cells reaching a new reference state for remodeling such that the changes in RANKL and OPG expression return to baseline level with high frequency 1 Hr loading.

There may be concerns that only mRNA levels were quantified and not cellular or physiological endpoints such as changes in protein levels, osteoclastogenesis, or osteoclast activity. There may also be concerns that alterations in RANKL/OPG ratio with varying flow frequencies may not necessarily regulate osteoclastogenesis in vivo. However, our previous studies on RANKL and OPG show that that these changes in mRNA are directly correlated to protein changes, osteoclast numbers, and osteoclastic pit formations.[13,38] Also, cells of osteoblast lineage are known to be vital in osteoclastogenesis and osteoclastic bone resorption. For example, cell-to-cell contact between osteoblastic/stromal cells and osteoclastic precursors are necessary in the fusion stage of osteoclast formation.[39] In addition, osteocyte ablation mice exhibit increased osteoclastic bone resorption and cortical porosity, decreased bone strength and trabecular bone volume, and defective mechanosensing ability demonstrating that importance of RANKL and OPG in the study of bone remodeling.[40]

## Conclusion

In summary, our results indicate that high frequency OFF of up to 10 Hz may accelerate the decrease in RANKL/OPG but through a different mechanism compared to low frequency 1 Hz loading. Although high frequency OFF beyond 10 Hz does not appear to further enhance the extent of decrease in the RANKL/OPG ratio, applying different loading frequencies appears to have the potential to differentially control the change in either RANKL or OPG mRNA expression.

## Competing interests

The authors declare that they have no competing interests.

## Authors' contributions

CHK carried out all experiments and drafted the manuscript. KHK participated in the interpretation of data and revised the manuscript. CRJ participated in the design of the study and revised the manuscript. All authors read and approved the final manuscript.

## Pre-publication history

The pre-publication history for this paper can be accessed here:


